# Robotic versus laparoscopic transabdominal preperitoneal (TAPP) approaches to bilateral hernia repair: a multicenter retrospective study using propensity score matching analysis

**DOI:** 10.1007/s00464-022-09614-y

**Published:** 2022-09-26

**Authors:** Roberto Peltrini, Francesco Corcione, Daniela Pacella, Simone Castiglioni, Ruggero Lionetti, Jacopo Andreuccetti, Giusto Pignata, Carlo De Nisco, Luca Ferraro, Adelona Salaj, Giampaolo Formisano, Paolo Pietro Bianchi, Umberto Bracale

**Affiliations:** 1 Department of Public Health, School of Medicine and Surgery, University of Naples Federico II, Via Pansini 5, 80131 Naples, Italy; 2 Department of General Surgery II, Spedali Civili of Brescia, Brescia, Italy; 3 General Surgery Unit, San Francesco Hospital, ASSL Nuoro, Nuoro, Italy; 4 Division of General and Robotic Surgery, Dipartimento Di Scienze Della Salute, University of Milano, Milan, ASST Santi Paolo E Carlo, Milan, Italy

**Keywords:** Laparoscopy, Robotic surgery, Inguinal hernia, Bilateral hernia, TAPP, Outcomes

## Abstract

**Background:**

Since the introduction of minimally invasive surgery, new techniques like transabdominal preperitoneal (TAPP) repair have progressively gained acceptance for the treatment of groin hernia. Laparoscopic TAPP (LTAPP) is recommended for bilateral repairs. Likewise, the introduction of robotic platforms has promised additional surgical benefits for robotic TAPP (RTAPP), which are yet to be confirmed. This study compared multicenter data obtained from patients undergoing bilateral inguinal hernia repair with RTAPP, performed during the preliminary learning curve period, versus conventional LTAPP.

**Materials and methods:**

All consecutive bilateral inguinal hernia patients from four Italian centers between June 2015 and July 2020 were selected. A propensity score model was used to compare patients treated with LTAPP versus RTAPP, considering sex, age, body mass index, current smoking status, overall comorbidity, hernia classification (primary or recurrent), and associated procedures as covariates. After matching, intraoperative details and postoperative outcomes were evaluated.

**Results:**

In total, 275 LTAPP and 40 RTAPP were performed. After matching, 80 and 40 patients were allocated to the LTAPP and RTAPP cohorts, respectively. No intraoperative complications or conversion to open surgery occurred. However, a longer operative time was recorded in the RTAPP group (79 ± 21 versus 98 ± 29 min; *p* < 0.001). Postoperative visual analog scale (VAS) pain scores (*p* = 0.13) did not differ and complication rates were similar. There were no clinical recurrences in either group, with mean follow-up periods of 52 ± 14 (LTAPP) and 35 ± 8 (RTAPP) months. A statistical difference in length of hospital stay was found between the groups (1.05 ± 0.22 vs 1.50 ± 0.74 days; *p* < 0.001).

**Conclusion:**

In this patient population, outcomes for bilateral inguinal hernia repair appear comparable for RTAPP and LTAPP, except for a shorter recovery after laparoscopic surgery. A longer operative time for robotic surgery could be attributable to the learning curve period of each center.

Inguinal hernia repair is one of the most performed procedures worldwide, with more than 20 million repairs performed each year [1, 2]. Since the introduction of minimally invasive surgery, transabdominal preperitoneal (TAPP) repair and totally extraperitoneal repair (TEP) have gained acceptance for the treatment of groin hernia (GH). The International Guidelines for Groin Hernia Management recommend laparoendoscopic repair for the treatment of primary bilateral GH, provided that a surgeon with sufficient and specific resources is available [3]. More recently, the introduction of robotic platforms has increased surgeons’ enthusiasm by claiming presumed benefits not only for more complex surgical procedures [4] but also for robotic TAPP (RTAPP) [5].

Only a few studies on robotic repair of GH have been published [5–7], frequently reporting single-center or single-surgeon experiences. In this regard, retrospective studies can often be affected by selection bias when comparing two different surgical approaches. Furthermore, data focusing on the treatment of bilateral inguinal hernias using a minimally invasive approach are scarce.

This study compared multicenter data collected from patients undergoing bilateral hernia repair with RTAPP, performed during the preliminary learning curve period, versus conventional LTAPP, to assess whether one approach is superior to the other in terms of perioperative outcomes.

## Materials and methods

All consecutive patients with bilateral inguinal hernias repaired by LTAPP and RTAPP were retrospectively selected from four Italian centers from June 2015 till July 2020. All participating surgeons had previously performed a minimum of 65 LTAPP procedures, completing the learning curve period [8]. The RTAPP was performed within a structured training program.

Patients in each cohort were matched for sex, age, body mass index (BMI), current smoking status, overall comorbidity, hernia classification (primary or recurrent), and associated procedures. Baseline demographic characteristics were obtained from a retrospective review of cases performed in each participating center. In addition, perioperative outcomes, including mean operative time, intraoperative details, postoperative complications, and recurrence rates, were investigated. All patients were invited to a standard clinical outpatient follow-up visit with their surgeon to assess for the development of seroma, hematoma, SSI, SSOPI, dysesthesia, chronic pain, or clinical recurrence. SSI was defined as an infection occurring in the surgical region and included superficial, deep, and organ space infections. SSOPI was a surgical site incident requiring a procedural intervention, defined as wound opening or debridement, suture excision, percutaneous drainage, or mesh removal [9].

### Surgical technique

#### Laparoscopic TAPP

Under general anesthesia, the patient was placed in the Trendelenburg position (30°) with the arms and legs adducted. The first 10 mm trocar was placed in the umbilicus, and two other 5 mm ports were placed bilaterally in the midclavicular line. After identification of the anatomical landmarks, the preperitoneal space was opened by incising the peritoneum transversely from the region of the umbilical artery laterally to the hernia defect. Dissection was performed in the Retzius and Bogros (retroinguinal) spaces. Sac dissection was performed carefully to safeguard the spermatic fascia and protect fragile parietal structures.

Different types of mesh (10 × 15 cm) were used, depending on availability. Except for Progrip, the mesh was securely fixed with 2 ml of fibrin glue (Tisseel/Tissucol, Baxter Healthcare, Deerfield, IL, USA). The peritoneum was closed with a running suture in V-Loc 3/0, and trocars were removed under direct visualization. The fascial defect of the 10 mm port was then closed.

#### Robotic TAPP

The patients were positioned in a similar manner to the LTAPP patients. To gain adequate working distance from the target anatomy, we used a supra-umbilical skin incision. Pneumoperitoneum was established using a Veress needle [10], and a non-bladed 8 mm trocar was inserted. Two robotic 8 mm trocars were inserted, one in the right upper quadrant and one in the left upper quadrant. These trocars were placed at a minimum of 8 cm lateral to the supra-umbilical port.

The dissection steps were performed in a manner identical to that of the laparoscopic approach. Once the contoured mesh was positioned, it was sutured in two locations with a 2-0 Vicryl: medially to Cooper’s ligament and superior-laterally to the abdominal wall. Fixation was not performed for cases using grip mesh. The peritoneal flap was closed with a running 2-0 barbed suture. If possible, the fascial defect was then closed.

### Statistical analysis

Data are presented as raw frequency and percentage for categorical variables and as mean ± standard deviation for continuous variables, with the corresponding 95% confidence interval. A propensity score model was implemented considering the following variables as covariates: sex, age, BMI, current smoking status, overall comorbidity, hernia classification (primary or recurrent), and associated procedure. Patients treated with the robotic approach and those treated with the laparoscopic approach were matched using nearest neighbor matching with a 1:2 ratio without replacement. The *χ*^2^ test and Fisher’s test were used to assess the differences among the LTAPP and RTAPP groups for categorical variables. The Student’s *t*-test or Mann–Whitney test, as appropriate, was used to analyze the differences among the LTAPP and RTAPP groups for continuous variables. The significance level for all analyses was set at *α* = 0.05. All analyses were performed using R statistical software version 4.0.3.

## Results

A total of 275 LTAPP and 40 RTAPP were performed. After matching, 80 and 40 patients were allocated to the LTAPP and RTAPP cohorts, respectively, with a mean age of 56 years and mean BMI of 25 in each group. Additionally, the comparative groups were homogeneous in terms of preoperative comorbidity rates and hernia type (75% primary and 25% recurrent hernias in both groups) (Table 1).

**Table 1:** Clinical data of each group after PSM

	LTAPP *n* = 80	95% CI	RTAPP *n *= 40	95% CI	*p*
Gender					> 0.999
Male	71 (89%)	(81.8,95.7)	35 (88%)	(77.3,97.8)	
Female	9 (11%)	(4.3,18.2)	5 (12%)	(2.3,22.8)	
Age (± SD)	56 (± 14)^a^	(52.3, 58.7)	56 (± 12)	(52.2, 60.0)	0.820
BMI (kg/m^2^)	25.54 (± 2.71)^a^	(24.9, 26.1)	25.37 (± 3.28)	(24.3, 26.4)	0.788
Current smoking	18 (22%)	(13.4,31.7)	12 (30%)	(15.8,44.2)	0.502
Alcohol abuse	0 (0%)	–	1 (2.5%)	(0.0,7.3)	0.333
Comorbidities					
Cardiovascular	32 (40%)	(29.3,50.7)	16 (40%)	(24.8,55.2)	> 0.999
Pulmonary	15 (19%)	(10.2,27.3)	9 (22%)	(9.6,35.4)	0.809
Urologic	16 (20%)	(11.2,28.8)	8 (20%)	(7.6,32.4)	> 0.999
Gastrointestinal	12 (15%)	(7.2,22.8)	9 (22%)	(9.6,35.4)	0.445
Current therapy					
Aspirin	14 (18%)	(9.2,25.8)	7 (18%)	(5.7,29.3)	> 0.999
Oral anticogulant	3 (3.8%)	(0.0,7.9)	3 (7.5%)	(0.0,15.7)	0.399
Steroid	0 (0%)	–	0 (0%)	–	> 0.999
Hernia classification					
Primary	60 (75%)	(65.5,84.5)	30 (75%)	(61.6,88.4)	> 0.999
Recurrent	20 (25%)	(15.5,34.5)	10 (25%)	(11.6,38.4)	> 0.999
Both lateral	42 (52%)	(41.6,63.4)	28 (70%)	(55.8,84.2)	0.102
Both medial	10 (12%)	(5.3,19.8)	5 (12%)	(2.3,22.8)	> 0.999
Lateral and medial	29 (36%)	(25.7,46.8)	7 (18%)	(5.7,29.3)	0.057

Data are reported as mean ± sd for continuous variables and as frequency (%) for categorical variables. 95% CI built around the means and the percentages are also reported.

^a^Mean value (± standard deviation)

No intraoperative complications or conversions to open or laparoscopic surgery occurred in either cohort of patients. However, a longer operative time was recorded in the RTAPP group (79 ± 21 min vs. 98 ± 29 min; *p* < 0.001).

Postoperative VAS pain scores (*p* = 0.13) and specific or overall complication rates did not differ between groups. In addition, there were no clinical recurrences in either group, over mean follow-up durations of 52 ± 14 (LTAPP) and 35 ± 8 (RTAPP) months (Tables 2 and 3). A statistical difference in length of hospital stay was found between the groups (1.05 ± 0.22 vs 1.50 ± 0.74 days; *p* < 0.001).

**Table 2:** Intraoperative data of each group after PSM

	LTAPP *n *= 80	95% CI	RTAPP *n* = 40	95% CI	*p*
Type of mesh					** < 0.001**
Ultrapro	38 (48%)	(36.6,58.4)	24 (60%)	(44.8,75.2)	
Progrip	4 (5.0%)	(0.2,9.8)	10 (25%)	(11.6,38.4)	
Parietex	36 (45%)	(34.1,55.9)	0 (0%)	–	
Softmesh	0 (0%)	–	0 (0%)	–	
Polipropilene	2 (2.5%)	(0.0,5.9)	6 (15%)	(3.9,26.1)	
Mesh size (cm)					0.204
15 × 10	77 (96.4%)	(88.5,99.1)	35 (88%)	(77.3,97.8)	
15 × 15	2 (2.5%)	(0.0,5.9)	1 (2.5%)	(0.0,7.3)	
20 × 15	1 (1.2%)	(0.0,3.7)	3 (7.5%)	(0.0,15.7)	
30 × 15	0 (0%)	–	1 (2.5%)	(0.0,7.3)	
Associated surgical procedures	12 (15%)	(7.2,22.8)	4 (10%)	(0.7,19.3)	0.635
Conversion to open surgery	0 (0%)	–	0 (0%)	–	–
Operative time (min)	79 (± 21)^a^	(74.5, 83.7)	98 (± 29)	(89.0, 107.3)	** < 0.001**
Length of stay (days)	1.05 (± 0.22)^a^	(1.0, 1.1)	1.50 (± 0.74)	(1.3, 1.7)	** < 0.001**

Data are reported as mean ± sd for continuous variables and as frequency (%) for categorical variables. 95% CI built around the means and the percentages are also reported.

Bold values denote statistical significance

^a^Mean value (± standard deviation)

**Table 3:** Postoperative outcomes of each group after PSM

	LTAPP *n* = 80	RTAPP *n* = 40
Overall complications	8 (10%)	5 (12.5%)
Seroma	5 (6.2%)	1 (2.5%)
Hematoma	2 (2.5%)	3 (7.5%)
SSI	1 (1.2%)	1 (2.5%)
SSOPI	0	0
Dysesthesia	0	0
Chronic pain	0	0
Intraoperative complications	0	0
Recurrence	0	0

## Discussion

Laparoscopic inguinal hernia repair is one of the most common procedures and is recognized as a safe, reliable, and feasible approach for inguinal hernia repair. In the European Hernia Society guidelines, the laparoscopic approach is recommended as one of the best evidence-based options by experts.

In recent years, the use of robotic platforms has gained popularity for various reasons: increased dexterity and improved engineering allow for easy maneuverability in performing a wide variety of procedures. The number of RTAPP procedures has progressively increased worldwide [11, 12], offering an increased range of instrument motion and improved surgeon ergonomics [5]. However, some concerns continue, including the operative time, surgeon’s learning curve, real patient effectiveness, and global costs [13, 14].

The present study shows the outcomes of using a robotic platform for bilateral inguinal transabdominal preperitoneal hernia repair compared to a laparoscopic approach in a multicenter setting. Propensity matching was performed to reduce selection bias. In our study population, we observed no intraoperative complications or conversions to open surgery; however, a longer operative time was recorded in the RTAPP group.

These results are consistent with those of a retrospective review of 63 patients who underwent RTAPP or LTAPP. The mean operative time (77.5 vs. 60.7 min, *p* = 0.001) and room time (109.3 vs. 93.0 min, *p* = 0.001) were significantly longer for the robotic vs the laparoscopic interventions [5]. The authors found that the robotic approach was certainly feasible, but it took significantly longer in terms of both operative time and room time when compared with the laparoscopic group. This was true for both unilateral and bilateral procedures. Although the robot provided excellent visualization and dissection in the preperitoneal space, the operative time was simply longer. Similarly, the only RCT [15] found that robotic transabdominal preperitoneal repair was associated with longer median operative times (75.5 [59.0–93.8] min vs. 40.5 [29.2–63.8] min, respectively; *p *< 0.001) than traditional laparoscopic inguinal hernia repair, although proficiency of participating surgeons using robotic platforms was assumed. Furthermore, the increased operative time for the robotic group was ascribed to each step of the operation; therefore, docking (median, 5 min) was not the culprit for the 35 min difference in time.

In contrast, Muysoms et al. [7] reported that RTAPP was associated with a rapid reduction in operative time over the learning curve; once a certain level of proficiency was achieved, the operative time to perform a RTAPP equaled the operative time to perform a LTAPP for both unilateral and bilateral GH repairs. Therefore, we emphasize that our results could be affected by a bias related to the learning curve. All surgeons involved in this study had already surpassed the adequate learning level for LTAPP [8], but were still relatively new to RTAPP.

There was no difference in postoperative VAS pain scores. A recent review reported that 11 studies, including the RIVAL trial [15], have compared pain scores of RTAPP and LTAPP. Perioperative and short-term pain did not differ in five of the six studies reporting this outcome [5, 7, 15–17] and one study detected less short-term pain after robotic surgery [18]. In terms of long-term pain, five of six studies found no difference between the two procedures [16, 19–22]; the remaining study observed an increased incidence of inguinodynia after RTAPP [23].

We found a statistically significant difference in length of hospital stay (LOS). Patients spent less time in recovery after LTAPP than RTAPP. These results are in contrast with those of previous studies that have not identified difference in LOS after laparoscopic or robotic approach for TAPP repair [5, 15]. Despite this slight but significant difference, the implications for the health system could be relevant even in the setting of future comprehensive cost-effective analyses. In fact, one of the most frequent topics in the use of robotic platforms is the higher cost compared to the laparoscopic approach. This issue has also been evaluated regarding the application of robotic platforms in other procedures [24]. Five previous studies reported cost information for inguinal hernia repairs [5, 6, 15, 25, 26]. All five studies compared the costs of robotic inguinal hernia repair to laparoscopic repair. The RIVAL trial [15] compared robot-assisted and laparoscopic transabdominal hernia repairs and found that the robot-assisted approach had more than twice the hospital cost of laparoscopy ($3258 [95% CI $2568–$4118] vs $1421 [95% CI $1196–$1930], *p* < 0.001). Notably, this study did not include capital equipment costs in its analysis, which would augment the cost differential between the two approaches. Of the remaining four observational studies, three found greater costs with robot-assisted inguinal hernia repair than with the laparoscopic approach, with a mean difference ranging from $2635 to $3999.

We recognize several limitations of this study. The retrospective nature led to missing data such as the defect size of the hernia that has not been considered as variable for matching. Large hernia defects have a significant impact on the occurrence of postoperative complications and conversion. Furthermore, we reported five patients with unusual large implanted meshes. However, mesh size have not been included in PSM. Both of these aspects may have affected the study outcomes.

The number of cases is definitely too small to be able to make statements about complications, therefore only a descriptive synthesis of the results was provided.

Finally, a possible statistical limitation of the present study is that, considering its exploratory nature, we did not perform corrections for multiple testing, which we plan to conduct in a future study involving a larger sample size.

## Conclusion

In this cohort of patients, the outcomes after RTAPP for bilateral inguinal hernia repair appear comparable to those after LTAPP, except for a shorter recovery after laparoscopic surgery. A longer operative time for robotic surgery could be attributable to the learning curve period of each center. The choice of the most suitable approach should be based on individual surgeon expertise and resource availability and should be tailored for each patient. From our results, as well as others from the literature, RTAPP does not seem to be a cost-effective alternative to LTAPP. However, we believe that RTAPP represents the best procedure for surgeons who begin their experience with robotic platforms, because it includes all the steps (dissection, reconstruction, and suturing) of more complex procedures.
